# Complete Genome Sequences of Three *Rhizobium gallicum* Symbionts Associated with Common Bean (*Phaseolus vulgaris*)

**DOI:** 10.1128/genomeA.00030-17

**Published:** 2017-03-16

**Authors:** Patricia Bustos, Rosa Isela Santamaría, Olga María Pérez-Carrascal, José Luis Acosta, Luis Lozano, Soledad Juárez, Irma Martínez-Flores, Esperanza Martínez-Romero, Miguel Ángel Cevallos, David Romero, Guillermo Dávila, Pablo Vinuesa, Fabiola Miranda, Ernesto Ormeño, Víctor González

**Affiliations:** Centro de Ciencias Genómicas, Universidad Nacional Autónoma de México, Cuernavaca, Morelos, México

## Abstract

The whole-genome sequences of three strains of *Rhizobium gallicum* reported here support the concept that the distinct nodulation host ranges displayed by the symbiovars gallicum and phaseoli can be largely explained by different symbiotic plasmids.

## GENOME ANNOUNCEMENT

*Rhizobium gallicum* is a nitrogen-fixing symbiotic bacterium of worldwide distribution that was initially isolated from common bean nodules (*Phaseolus vulgaris*). Here, we provide the complete genome sequences of two broad nodulation host range *R. gallicum* sv. *gallicum* strains: R602 and IE4872, and for the *R. gallicum* sv. *phaseoli* 8C-3 strain that nodulates only common bean. R602 is the type strain isolated in Maine-et-Loire in France by Amarger et al. (1). The IE4872 and 8C-3 strains were isolated in San Miguel Acuexcomac, Puebla, Mexico (2, 3), and Hornachuelos, Andalusia, Spain (4), respectively.

Genomic DNA was purified with QIAamp DNA Mini Kit, Qiagen. Illumina Miseq and 454 (Mogene LC, St. Louis MO, USA) technologies were used for DNA sequencing of libraries with inserts of about 200 bp, 2 kb and 3 kb in length; paired-end reads were assembled using Newbler 2.5.3 (Roche), Velvet 1/1/06 (5), Sspace-Basic 2.0 (6), and Consed v 23 (7), with a coverage between 150× and 400×. The complete genome assembly of the R602 strain was used as a reference to finish the IE4872 and 8C-3 genomes, first by performing a whole-genome pairwise alignment with the MUMmer program (8), and second, by manual and computational curing of the assemblies, using Consed. Open reading frames (ORFs) were predicted with Glimmer 3 (9); corrections of frameshifts and manual annotations were done in Artemis 12.0 (10), with comparisons with Genbank (11), Interpro (12), and ISfinder (www-is.biotoul.fr) databases.

The *R. gallicum* genomes are about 7.3 to 7.4 Mb in size, with overall G+C content of 60%. They consist of a 4.1-Mb chromosome and three to four large plasmids (2, 3); one of them encodes most of the symbiosis genes (i.e., the symbiotic plasmid pSym). The average nucleotide identities (ANI) of the whole genome of the R602 strain with respect to IE4872 and 8C-3 are about 96% and 93%, respectively. A common large plasmid between 2.25 and 2.46 Mb is present in the three strains. Another small plasmid (0.17 to 0.2 Mb) is present only in R602 and IE4872 strains, but not in 8C-3. The pSym plasmids of strains R602 and IE4872 are quite similar (ANIm of 97%) over 63% of the length of the replicon, whereas the pSym of the 8C-3 strain is very similar to the pSym found in *R. etli* CFN42 and *R. phaseoli* CIAT652. Comparisons of the *nod* gene set of *R. gallicum* sv. gallicum and phaseoli symbiotic plasmids indicate key differences in the protein gene products for sulfation (NodHPQ), carbamoylation (NodU), and in the α,β unsaturation of the lipid tail at the nonreducing end (NodEF, NodA2) of the Nod factor. These differences might be associated with the extended host range in gallicum strains.

Analysis of the complete genome sequences of different symbiovars will facilitate the identification of specific genes for determining host range (13) and the genetic basis for adaptations to distinct leguminous plant species.

### Accession number(s).

The complete genome sequences for the three *R. gallicum* strains have been deposited in NCBI Genbank under the sequence accession numbers CP006877 to CP006880, CP017101 to CP017105, and CP017241 to CP017244, for strains R602, IE4872, and 8C-3, respectively.
